# Light-induced formation of dimeric LHCII

**DOI:** 10.1007/s11120-017-0387-6

**Published:** 2017-04-19

**Authors:** Ewa Janik, Joanna Bednarska, Karol Sowinski, Rafal Luchowski, Monika Zubik, Wojciech Grudzinski, Wieslaw I. Gruszecki

**Affiliations:** 1 Department of Biophysics, Institute of Physics, Maria Curie-Sklodowska University, Pl. Marii Curie-Sklodowskiej 1, 20-031 Lublin, Poland; 2 Department of Cell Biology, Institute of Biology and Biochemistry, Maria Curie-Sklodowska University, ul. Akademicka 19, 20-033 Lublin, Poland; 3 Department of Medicine, Imperial College London, Du Cane Road, London, W12 0NN UK; 4 Chair and Department of Synthesis and Chemical Technology of Pharmaceutical Substances, Faculty of Pharmacy, Medical University, ul. Chodzki 4a, 20-093 Lublin, Poland; 5 Department of Metrology and Modelling of Agrophysical Processes, Institute of Agrophysics of Polish Academy of Sciences, ul. Doswiadczalna 4, 20-290 Lublin, Poland

**Keywords:** LHCII complex, Dimer, Photoprotection, Fluorescence quenching, *Spinacia oleracea*

## Abstract

It emerges from numerous experiments that LHCII, the major photosynthetic antenna complex of plants, can appear not only in the trimeric or monomeric states but also as a dimer. We address the problem whether the dimeric form of the complex is just a simple intermediate element of the trimer–monomer transformation or if it can also be a physiologically relevant molecular organization form? Dimers of LHCII were analyzed with application of native electrophoresis, time-resolved fluorescence spectroscopy, and fluorescence correlation spectroscopy. The results reveal the appearance of two types of LHCII dimers: one formed by the dissociation of one monomer from the trimeric structure and the other formed by association of monomers into a distinctively different molecular organizational form, characterized by a high rate of chlorophyll excitation quenching. The hypothetical structure of such an energy quencher is proposed. The high light-induced LHCII dimerization is discussed as a potential element of the photoprotective response in plants.

**Electronic supplementary material:**

The online version of this article (doi:10.1007/s11120-017-0387-6) contains supplementary material, which is available to authorized users.

## Introduction

Green plants, algae, and some species of bacteria utilize light energy to power photosynthetic reactions. It is known that the yield of photosynthesis depends strongly on the intensity of absorbed light. In the natural environment, the light conditions vary greatly in intensity and quality on a broad time scale, ranging from minutes up to hours, days, and even months. These changes can result from environmental factors such as cloud movement, sun flecks, weather system, or sudden shading by other plants and changes in the canopy structure (Owens 1994). Exposure of plants to short- as well as long-term high light illumination frequently leads to overexcitation of the reaction center of photosystems causing photoinhibition, particularly of Photosystem II (PSII). This phenomenon leads to irreparable damage of the D1 polypeptide, photosynthetic pigments and unsaturated fatty acids of thylakoid membranes (Marshall et al. 2000).

As a result of biological evolution, plants have developed diverse physiological responses that enable optimization of photosynthetic processes. These regulatory mechanisms, that act to adjust excitation density in the photosynthetic apparatus to the current capacity of photochemical reactions, operate at different organizational levels of plant organisms, beginning with the phototropic movement of entire leaves (Koller 1990), through photo-translocation of chloroplasts within a cell (Chow et al. 1988), ending with supramolecular reorganization of pigment–protein complexes of both photosystems at the molecular level (Ruban 2009). The significant role in photoprotection is attributed to the major light-harvesting pigment–protein complex of PSII (LHCII). Its native and functional form is a trimer (Liu et al. 2004; Ruban et al. 2003; Wei et al. 2016; Barros and Kuhlbrandt 2009). Each monomer of the trimer is composed of a polypeptide of about 232 aminoacid residues, constituting three transmembrane and two short α-helices. The polypeptide chain binds 14 molecules of chlorophyll: eight molecules of chlorophyll *a* (Chl *a*) and six molecules of chlorophyll *b* (Chl *b*). Additionally, the complex comprises four xanthophyll molecules: one violaxanthin (or zeaxanthin), one neoxanthin, and two luteins (Liu et al. 2004; Standfuss et al. 2005). The dynamic regulation of light-harvesting efficiency is responsible for the balance between absorption and utilization of light energy and therefore protects the reaction centers against photodamage. For a number of years, evidence has accumulated that changes in the molecular organization of LHCII affect yield of energy excess quenching. It was proposed that LHCII aggregates are direct quenchers of excess excitation (Horton et al. 1991; Magdaong et al. 2013; Johnson and Ruban 2009a). The most recent study on intact chloroplast and isolated LHCII complexes evidenced that the trimeric structure of LHCII is exceptionally well adjusted to light quanta absorption (Janik et al. 2015) and the LHCII monomer, which is less efficient in excitation energy transfer, could be potentially effective in dissipation of excess energy. It has been noticed that light-driven trimer to monomer transition of the complex could regulate the physiological role of the antenna complexes in the thylakoid membrane (Janik et al. 2015; Garab et al. 2002). Naturally, the process of the trimer to monomer transformation of LHCII can potentially involve an intermediate step which is a formation of a dimeric structure. Indeed, the formation of LHCII dimeric forms has been recently observed as induced by interaction of the complex with zeaxanthin which was concluded to weaken inter-monomer interactions within a LHCII trimer (Janik et al. 2016). Interestingly, the results of the recent studies of Xu et al. reveal that zeaxanthin engaged in protection of the photosynthetic apparatus against strong light-induced damage is bound at the antenna complexes periphery rather than in the specific xanthophyll-binding sites (Xu et al. 2015). Such a finding corroborates with a scenario that reorganization of trimeric LHCII is associated with the photoprotective response in the photosynthetic apparatus. In the present work, we address the problem whether, the dimeric form of the complex, induced by illumination of LHCII preparation containing trimers, is just a simple intermediate element of a trimer–monomer transformation or rather, a molecular organization form of the complex which can play certain physiological functions.

## Materials and methods

### LHCII isolation

Fresh leaves of *Spinacia oleracea* (L.) were purchased from the local market. Before LHCII isolation the spinach leaves were kept at 4 °C in the dark for 24 h in order to induce starch depletion. Dark-acclimated leaves were exposed to high light intensity of 1200 µmol photons m^−2^ s^−1^ for 0.5 h. The LHCII complexes were isolated from spinach leaves that were dark-adapted or illuminated with high light according to the methods described in (Krupa et al. 1987) or (Janik et al. 2013), respectively. The purity of preparations was determined using HPLC and electrophoresis (Gruszecki et al. 2009). The molar ratio of xanthophyll pigments (determined for two molecules of lutein) in the dark-preparation was as follows: violaxanthin 0.56 ± 0.04, neoxanthin 1.04 ± 0.03, zeaxanthin—not detected, and in the high light-treated preparation was: violaxanthin 0.18 ± 0.01, neoxanthin 0.93 ± 0.03, zeaxanthin—not detected. Fraction determination of oligomeric forms of LHCII (trimers, dimers and monomers) in both preparations was carried out using the non-denaturing gel electrophoresis method described below. The Chl *a*/Chl *b* molar ratio in both preparations was 1.33 ± 0.06. The chlorophyll concentrations were calculated accordingly to (Lichtenthaler 1987).

### Sample preparation

#### LHCII in detergent solution

LHCII complexes (isolated from dark-adapted spinach leaves) were centrifuged at 15,000*g* for 5 min. Next, the pellet was dissolved in tricine buffer (20 mM Tricine, 10 mM KCl, pH 7.6) containing *n*-Dodecyl-β-d-maltoside (DM). DM was used in two concentrations: 0.1 and 0.03% in order to ensure stability of a given LHCII molecular organization form: mixture of LHCII monomers and trimers (0.1% DM) and suspension of LHCII trimers (0.03% DM). Next, the LHCII samples were vortexed for 30 min. After incubation with DM, LHCII complexes were centrifuged at 15,000*g* for 5 min to separate remaining aggregated forms. Reconstituted LHCII monomers were refolded and purified on a sucrose density gradient centrifugation according to the method described elsewhere (Ruhle and Paulsen 2011).

#### Lipid–LHCII with exogenous zeaxanthin

LHCII samples enriched with exogenously added zeaxanthin were prepared as described previously (Janik et al. 2016). The LHCII samples were separated using “native” electrophoresis as described below. After separation, gel strips with zeaxanthin-induced dimeric forms of LHCII were cut and collected for spectroscopic measurements.

#### LHCII complexes in a polyacrylamide gel strips

LHCII trimers and monomers were prepared from the dark LHCII preparation by non-denaturing “native” gel electrophoresis. Light-induced LHCII trimers, dimers, and monomers were derived by illumination of the LHCII samples located in electrophoretic slots during the electrophoretic separation. Each slot was illuminated with different light intensity (from ~10 to ~1200 µmol photons m^−2^ s^−1^). It was achieved by gel illumination in an intensity gradient of a LED light source (schematic presentation in Fig. S1). Light intensity in each slot was precisely measured using a photometer (FR 10 OPTEL, Poland) directly before each experiment. All the LHCII samples were electrophoresed in a 3% stacking and 8% separating polyacrylamide gels. The LHCII samples (140 µg/ml of chlorophyll *a* + *b*) were mixed with 60% sucrose (2:1, v:v) and loaded per lane of 1 mm thick gel. The separation was performed at a constant current of 4 mA per gel for approximately 1.5 h at 4 °C in running buffer (96 mM Glycine, 12.5 mM Tris, 0.05% LDS). The chlorophyll content of the green bands was analyzed quantitatively using the ImageJ2x image analysis software. After electrophoretic separation, gel strips containing LHCII complexes were cut and used in spectroscopic measurements. The gel thickness was 1 mm. The various LHCII oligomeric forms located in a polyacrylamide gel were studied previously (Ilioaia et al. 2008; Janik et al. 2015, 2016). The pigment composition and spectroscopic characteristic of the LHCII trimers and monomers obtained by the method described above were presented in (Janik et al. 2015, 2016) and show that the native gel methods yields fully functional complexes.

### Spectroscopic measurements

#### Circular dichroism spectroscopy

CD spectra were detected on a Chirascan-plus spectrometer (Applied Photophysics, UK) in the range of 300–800 nm, at room temperature. The spectra were measured from the LHCII complexes located in a polyacrylamide gel. Gel slices of 1 mm thickness were sandwiched between glass plates.

#### Steady-state fluorescence spectroscopy

Spectra of Chl *a* fluorescence emission were detected using Cary Eclipse spectrofluorometer (Varian, Australia). The excitation wavelength was set at 470 nm. The measurements were performed at 77 K. The spectra were measured from the LHCII complexes located in a polyacrylamide gel.

#### Time-resolved fluorescence spectroscopy

Fluorescence lifetime of LHCII trimers, dimers, and monomers were measured using FluoTime 300 spectrometer (PicoQuant, Germany). Excitation was set at 470 nm from a solid state LDH-P-C-470 laser with 20 MHz frequency of pulses and with a pulse width of 70 ps. Detection (at 680 nm) was done with a micro-channel plate and time-correlated single-photon counting system PicoHarp 300. Fluorescence decay curves were fitted with FluoFit Pro v 4.5.3.0 (PicoQuant, Germany). Fluorescence intensity decays were analyzed by reconvolution with the instrument response function and analyzed as a sum of exponential terms. The quality of the fit was judged from the χ^2^ value.

#### Fluorescence Correlation Spectroscopy (FCS)

LHCII in 0.1% DM solution (preparation procedure described above) was illuminated with light intensity of 100 µmol photons m^−2^ s^−1^ (halogen illuminator combined with a band-pass interference filter centered at 450 nm [band width 80 nm, Melles Griot, USA]). Next, the LHCII solution was diluted to a 3 nM concentration and subjected to measurements. Monomers and trimers of LHCII were prepared as described above. Diffusion coefficients of LHCII oligomers were determined on the basis of fits to the experimental fluorescence correlation spectroscopy curves. FCS measurements were performed on a confocal MicroTime 200 (PicoQuant, GmbH, Germany). The system was coupled to an Olympus IX71 microscope. The sample, at nanomolar concentration, was placed on non-fluorescent glass slides (Menzel-Glaser) on the microscopic table. Excitation was obtained by a 405 nm pulsed laser with a repetition rate of 20 MHz. The FWHM of the pulse response function was 68 ps (measured by PicoQuant, Inc.). The laser beam was collimated in the scan head and focused using ×60 water immersed objective (NA 1.2, Olympus) on to a sample volume. In order to select a single focal plane and reduce excitation light a pinhole with a diameter of 30 and 50 µm, was used. Time resolution was 4 ps. The observation was made through 680/13 band-pass filter. Scattered light was removed using ZT 473 RDC dichroic filter (Analysentechnik). Fluorescence photons were collected with a single-photon sensitive avalanche photodiode (APD) with processing accomplished by the HydraHarp400 time-correlated single-photon counting (TCSPC) module. Data analysis was performed using SymPhoTime 64 software package. The correlation function for three-dimensional diffusion was calculated as:

1 $$G\left( \tau \right)=G\left( 0 \right) \times {\left( {1+\frac{\tau }{{{\tau _{\text{D}}}}}} \right)^{ - 1}} \times {\left( {1+{{\left( {\frac{s}{u}} \right)}^2} \times \frac{\tau }{{{\tau _{\text{D}}}}}} \right)^{ - \frac{1}{2}}},$$

where *G*(0) is the amplitude at *τ* = 0, *τ* is time, *τ*_D_ is diffusion time, *s* is the radius, and *u* is the half-length of the ellipsoidal confocal volume. *τ*_D_ gives the diffusion coefficient:

2 $$D=\frac{{{s^2}}}{{4{\tau _{\text{D}}}}}$$

## Results and discussion

**Fig. 1:**
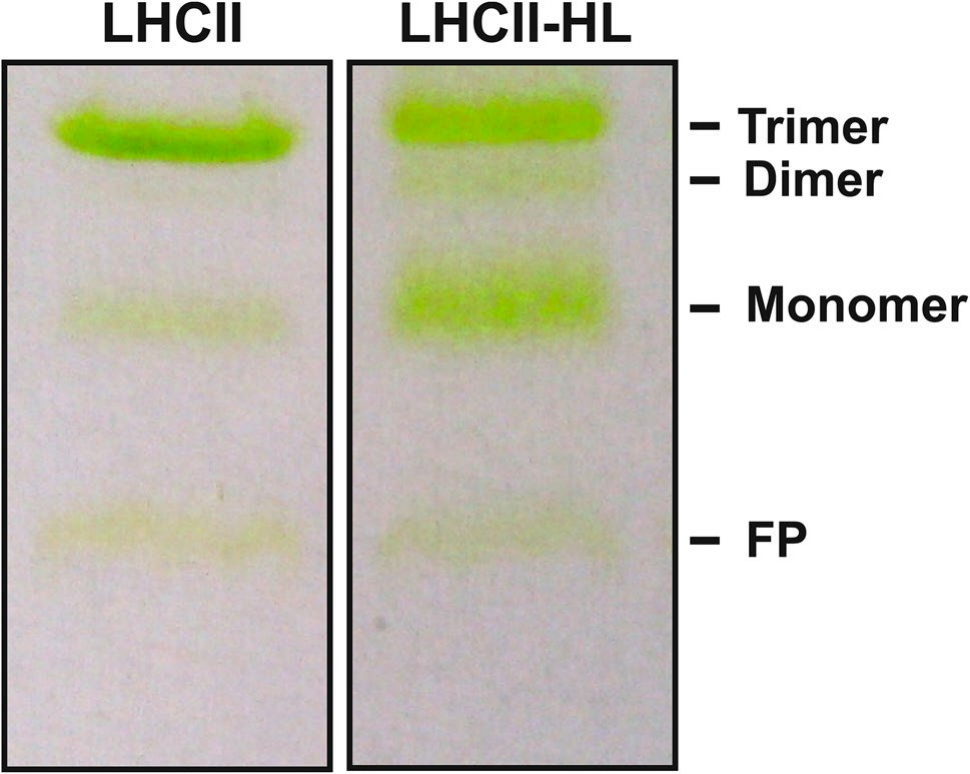
Electrophoretic analysis of LHCII oligomeric forms in LHCII preparations isolated from *Spinacia oleracea* leaves. Spinach leaves were dark-adapted (LHCII) or pre-illuminated with light intensity of 1200 µmol photons m^−2^ s^−1^ (LHCII-HL) before LHCII isolation. LHCII was suspended in 0.1% DM. Figure shows exemplary results of the electrophoretic gel analyses with band assignment (*FP* free pigments fraction)

According to our previous observations, appearance of a dimeric form of LHCII can be discerned in process of zeaxanthin-induced trimer disassembling (Janik et al. 2016). Additionally, the dimeric form of LHCII complexes can be observed in LHCII preparations isolated from spinach leaves, treated with high light intensity. Figure 1 presents an electrophoretic separation of LHCII preparations isolated from spinach leaves, dark-adapted (LHCII) or pre-illuminated with high light intensity (LHCII-HL, 1200 µmol photons m^−2^ s^−1^). LHCII samples isolated from the dark-adapted leaves mainly contain the trimeric form of LHCII (trimer/monomer ratio = 2.82 ± 0.41), unlike the LHCII-HL sample consisting of trimers, a relatively large pool of monomers (trimer/monomer ratio = 0.71 ± 0.09) and also dimers. A similar high light-induced decrease in the trimer/monomer ratio was shown earlier on isolated pea LHCII complexes, PSII-enriched spinach grana membranes, intact thylakoid membranes, and a whole Arabidopsis plants (Garab et al. 2002). Moreover, Standfuss and Kühlbrandt recorded dimer as a structure accompanying a large pool of Lhcb3 monomers when oligomers of Lhcb3 isoform were studied (Standfuss and Kuhlbrandt 2004). Both, LHCII and LHCII-HL samples, analyzed in our experiment, were isolated from spinach leaves in the same biochemical steps. Moreover, Bielczynski et al. (2016) verified the high stability of the trimers to the detergent treatment. So, it can be concluded that a large population of the LHCII monomers, and therefore existence of dimers in the LHCII-HL sample is not an artifact caused by the isolation procedure.

**Fig. 2:**
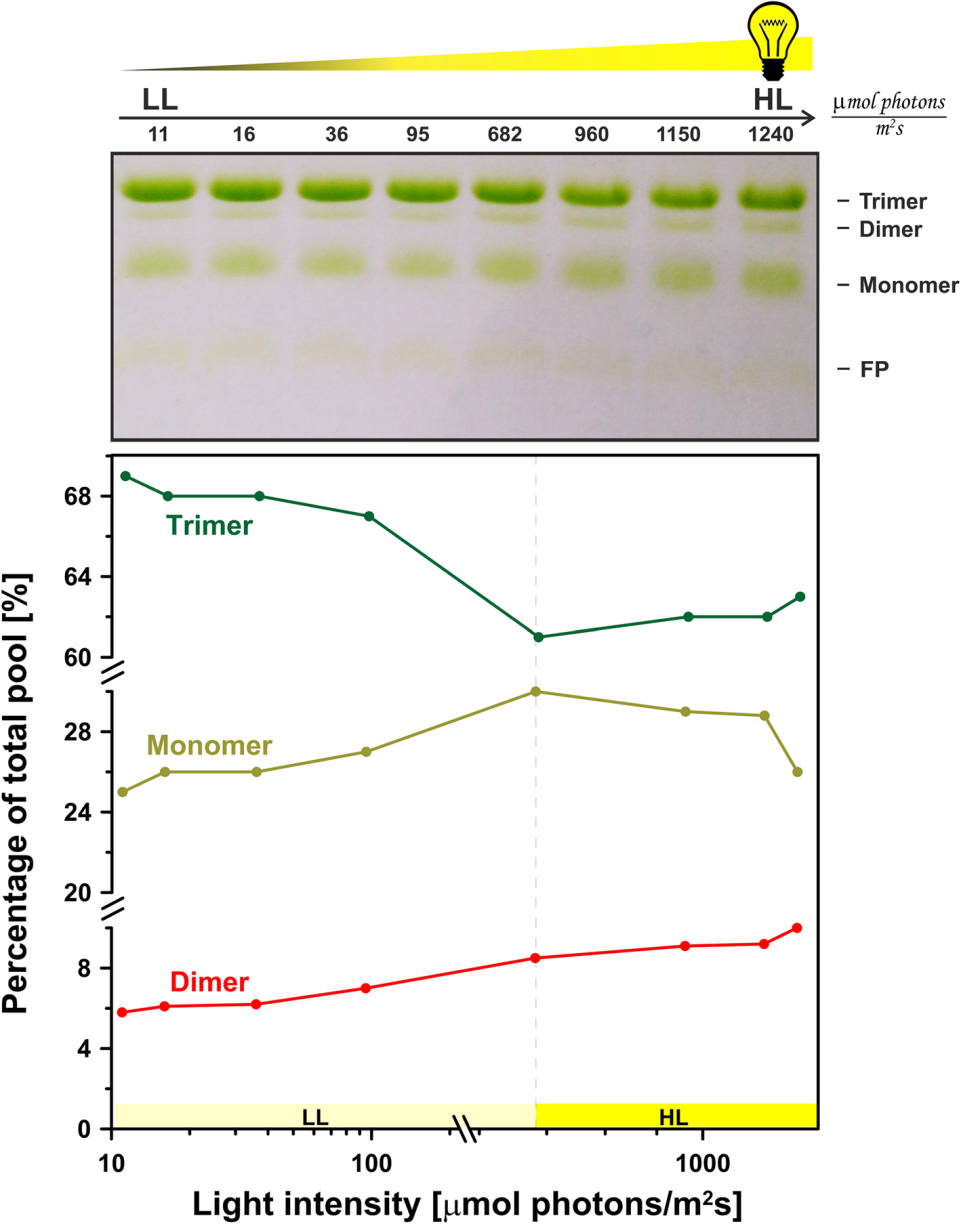
Electrophoretic analysis of LHCII oligomeric forms in LHCII samples illuminated with different light intensities. LHCII was suspended in 0.1% DM. Electrophoresis was conducted under constant illumination starting from the sample loading. Each slot was illuminated with different light intensity (from 11 µmol photons m^−2^ s^−1^ to 1240 µmol photons m^−2^ s^−1^). This was carried out by gel illumination in an intensity gradient of a LED light source (as it is schematically presented in Fig. S1). Light intensity in each slot was precisely measured using a photometer before commencing each experiment. *Upper panel* shows exemplary results of the electrophoretic gel analyses with band assignment (*FP* free pigments fraction). *Lower panel* presents the quantity analysis of LHCII trimers, dimers, and monomers in the samples. Relative intensities of the bands corresponding to different LHCII forms were quantified by scans analyzed with ImageJ2x software. Experiment was repeated ten times. Similar, exemplary effect of different light intensities on the LHCII trimers and monomers mixture is presented as Supplementary data (Fig. S2)

In order to check whether LHCII dimers could be directly induced by high light, an experiment was performed with 0.1% DM LHCII (preparation isolated from dark-adapted leaves) suspension illuminated with light intensities from the range of 11–1240 µmol photons m^−2^ s^−1^. Figure 2 presents the results of an electrophoretic separation of LHCII illuminated directly after loading onto the stacking gel. As can be seen in the lower panel of Fig. 2, in the light intensity range of 11–95 µmol photons m^−2^ s^−1^, the pool of LHCII trimers decreased and simultaneously the amount of monomers and dimers increased. In order to verify if such a process is typical only of the isolated LHCII suspension, an identical experiment with the thylakoid membranes isolated from the dark-adapted spinach leaves was performed. A relatively low detergent (0.1% DM) was chosen for thylakoid membrane solubilization based on the observation that high light-induced LHCII dimers are not stable when exposed to a higher detergent concentration (Fig. S3). The electrophoretic pattern of the pigment–protein complexes obtained for illuminated detergent suspension of thylakoids changed along with increasing light intensity (Fig. S4). It is important to our discussion that a decrease in the amount of the LHCII trimers was associated with an increase in a fraction of monomeric antenna complexes under high light treatment. Moreover, band localized between the trimeric and the monomeric antenna forms was detected. Interestingly, intensity of this band increased along with increasing light intensity. Therefore, on the basis of the above results, we can conclude that the light-induced formation of dimeric structures of LHCII can also take place in detergent suspension of the thylakoid membranes. An observed increase in the LHCII monomers pool can be interpreted in terms of the LHCII trimer to monomer transition occurring at moderate light intensities (Janik et al. 2015). It has been proposed that a thermo-optic effect is responsible for this photo-transformation (Garab et al. 2002). Under the same conditions, a light-induced *trans*–*cis* molecular configuration change of violaxanthin was reported (Grudzinski et al. 2001). Based on the results of the Raman analysis, this latter effect has been hypothesized as directly associated with the light-induced trimer to monomer transition (Gruszecki et al. 2009). It is possible that both the mechanisms of the thermo-optic effect and violaxanthin photo-isomerization act synergistically to activate light-induced LHCII trimer to monomer transformation. It is clearly visible that the amount of LHCII dimers is dependent on light intensity, the higher the photon flux density the more dimers present (Fig. 2). Surprisingly, at higher light intensities (682–1240 µmol photons m^−2^ s^−1^), increase in the concentration of LHCII dimers is associated with a decrease in the fraction of monomers rather than with the decrease in the fraction of trimers. Such a behavior suggests that dimeric forms of LHCII can arise not only as a result of the disassembly of trimers but also through association of monomers. It must be noted that monomers in a detergent solution can interact in the upside-down orientation that is not possible in vivo in the thylakoid membrane environment. However, it should be considered that dimers observed in the LHCII preparation isolated from the high light-illuminated spinach leaves (Fig. 1) were formed in the thylakoid membranes where the monomers are oriented the same way.

**Fig. 3:**
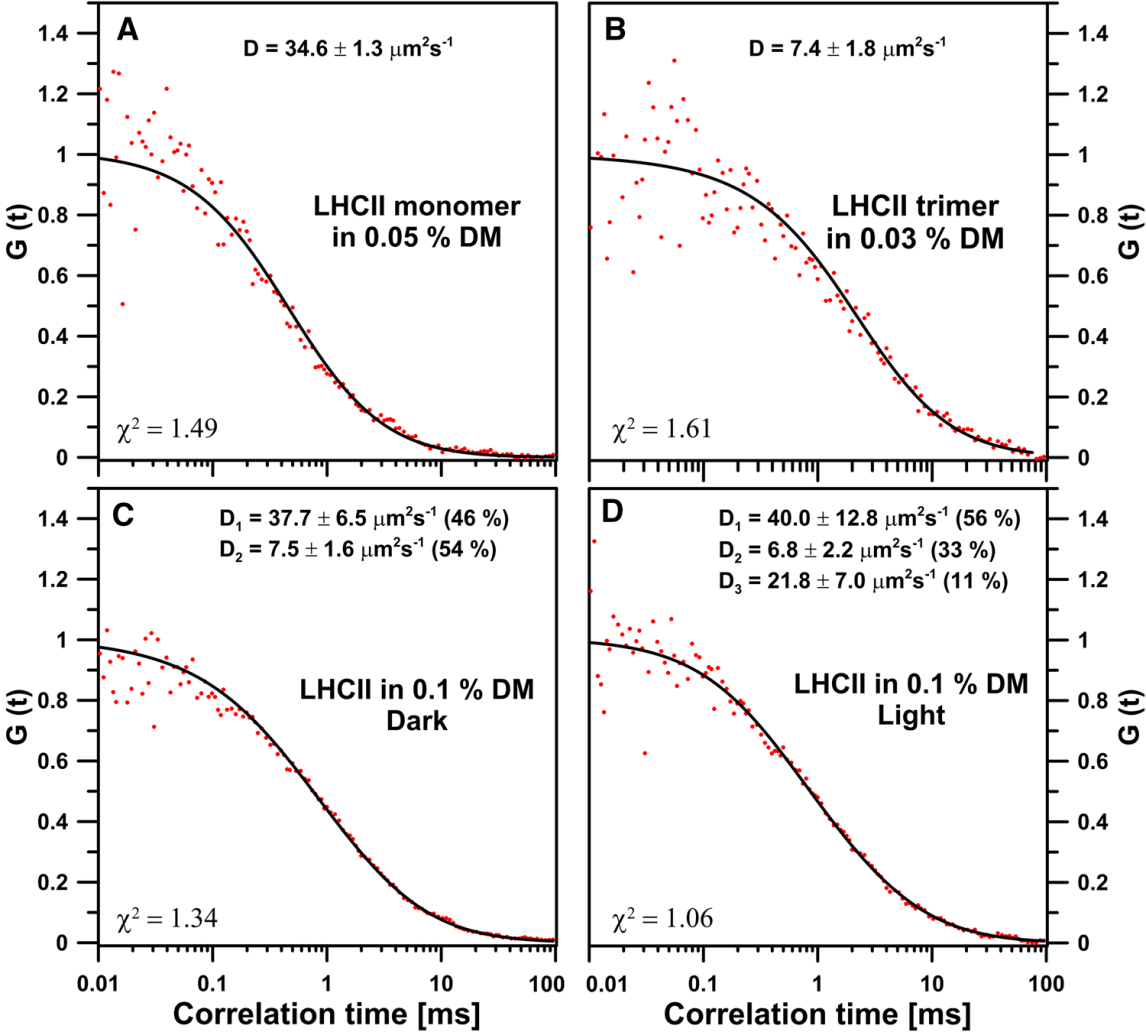
Normalized autocorrelation curves of different LHCII forms. Monomers (**a**) and trimers (**b**) were suspended in 0.05 and 0.03% DM solution, respectively. Dark-adapted LHCII trimers and monomers mixture in 0.1% DM solution (**c**) were subjected to illumination with blue light intensity of 100 µmol photons m^−2^ s^−1^ for 30 min (**d**). Diffusion coefficient values [as mean values ± SD (*n* = 8 biological replicates)] of the LHCII forms detected during FCS measurements are presented in the figure

Formation of LHCII dimeric structures can be observed in detergent solution not only in the environment of an electrophoretic separation. Such a conclusion is based on the results of the Fluorescence Correlation Spectroscopy (FCS) analysis (Fig. 3). Measurements were based on temporal fluctuations of Chl *a* fluorescence intensity resulting from the spontaneous diffusion of LHCII complexes in a detergent (DM) solution. In principle, the FCS method allows detection of a single fluorescent molecules passing through very small observation volume (in our experiment ~0.4 fl) and the determination of said molecular parameters, such as the diffusion coefficient (D) (Magde et al. 1972, 1974; Elson and Magde 1974). This method was used to study the diffusion coefficient of pigment–protein complexes in thylakoid membranes isolated from *Chlamydomonas reinhardtii* (Iwai et al. 2013). In our experiment, diffusion coefficients of pure trimeric and monomeric forms of LHCII in DM solution were estimated and used as a standard control for measurements of the diffusion coefficient of the LHCII oligomeric forms induced by light. LHCII monomers used in this study were reconstituted without phosphatidylglycerol molecules in order to prevent spontaneous trimerization of LHCII during the measurements. Based on the analyses of the samples dominated by the protein monomers (Fig. 3a) and trimers (Fig. 3b), the diffusion coefficients for those forms can be assigned values of approximately to 35 and 7 µm^2^ s^−1^, respectively. Illumination of a mixture of the trimeric and monomeric LHCII forms in 0.1% DM solution (Fig. 3c) results in the appearance of an additional molecular organization form characterized by a diffusion coefficient at a level of 22 µm^2^ s^−1^ (Fig. 3d), which can be assigned to the dimeric form of LHCII. Such an observation and the presence of the LHCII dimers in the preparation isolated from the high light-treated leaves (Fig. 1) suggest that the LHCII dimers observed during illumination of the electrophoretic separation were not a degradation product of the electrophoresed LHCII. Additionally, supported by the fact that the appearance of dimers reduces the monomeric fraction only during electrophoresis. What is more, isolation of dimers from the high light-illuminated spinach leaves strongly implies formation of those structures in vivo.

**Fig. 4:**
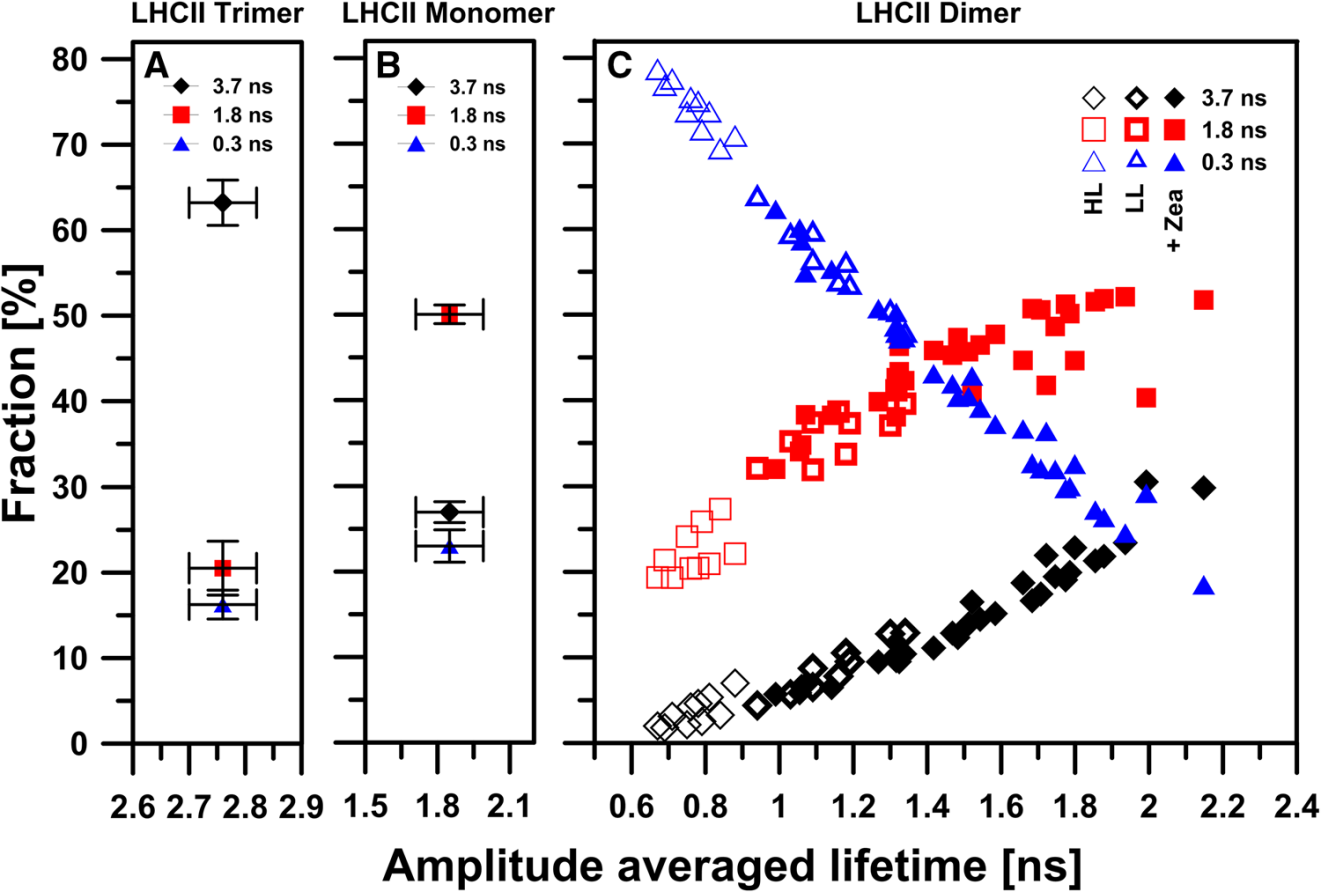
Amplitude-weighted average lifetime <*τ*> values in relation to amplitude of lifetime components measured from LHCII trimer, dimer, and monomer. Values of average lifetime were calculated on the basis of the fluorescence decay kinetics fitted with three components characterized by the following lifetimes: *τ*_1_ = 3.7 ns, *τ*_2_ = 1.8 ns and *τ*_3_ = 0.3 ns. Average lifetimes for the LHCII trimers and monomers are presented as mean values. *Error bars* indicate standard deviation (*n* = 10 biological replicates). Fluorescence intensity decays were analyzed by deconvolution with the instrument response function and analyzed as a sum of exponential terms. The quality of the fit was judged from the χ^2^ value (~1). Excitation and detection was at 470 and 680 nm, respectively. Fluorescence decay curves were recorded from LHCII complexes located in polyacrylamide gel separated by means of the non-denaturing gel electrophoresis

**Fig. 5:**
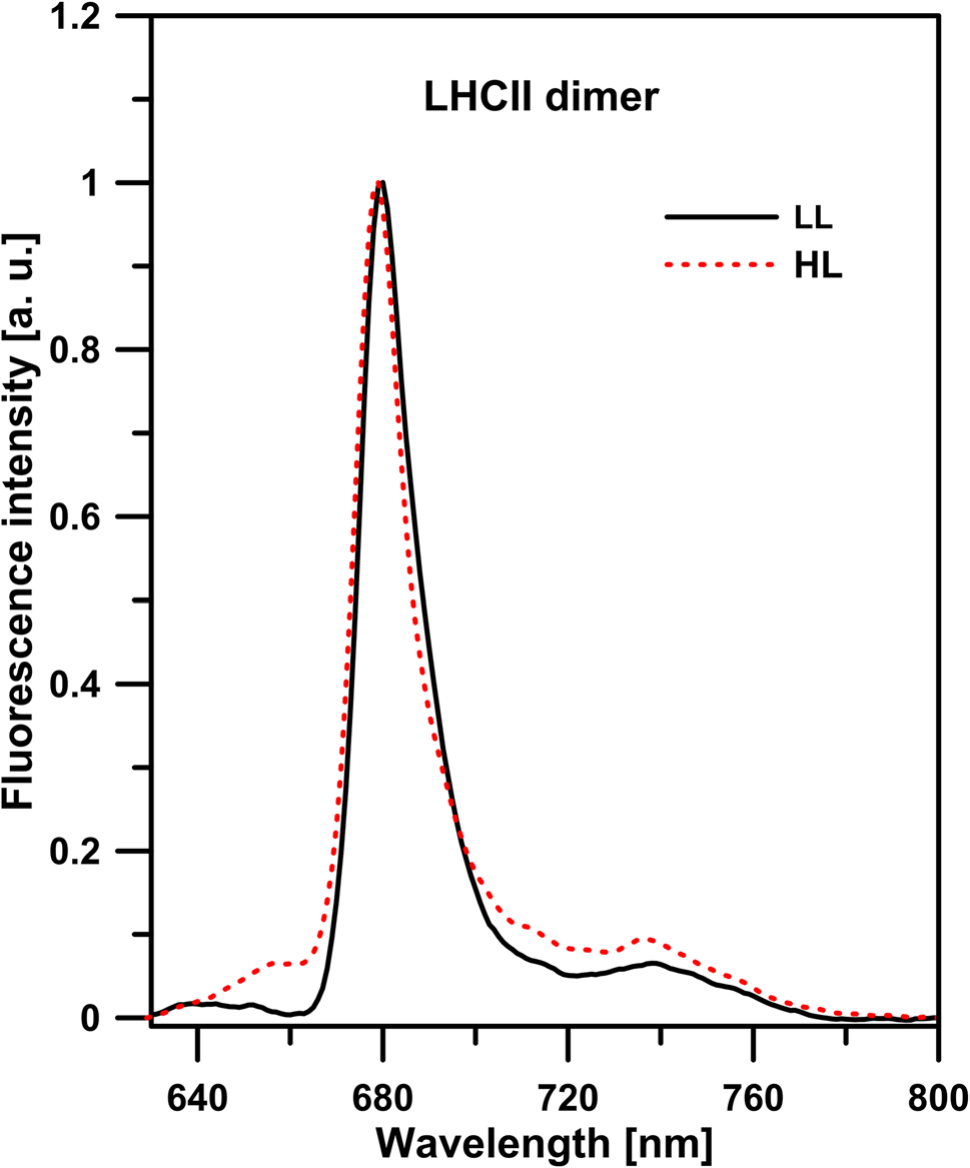
77 K Chl *a* fluorescence emission spectra of the LHCII dimers. Dimers were induced by low (18 µmol photons m^−2^ s^−1^) or high (1200 µmol photons m^−2^ s^−1^) light intensity. The spectra were normalized in the maximum. Spectra were measured from the LHCII complexes located in polyacrylamide gel

**Fig. 6:**
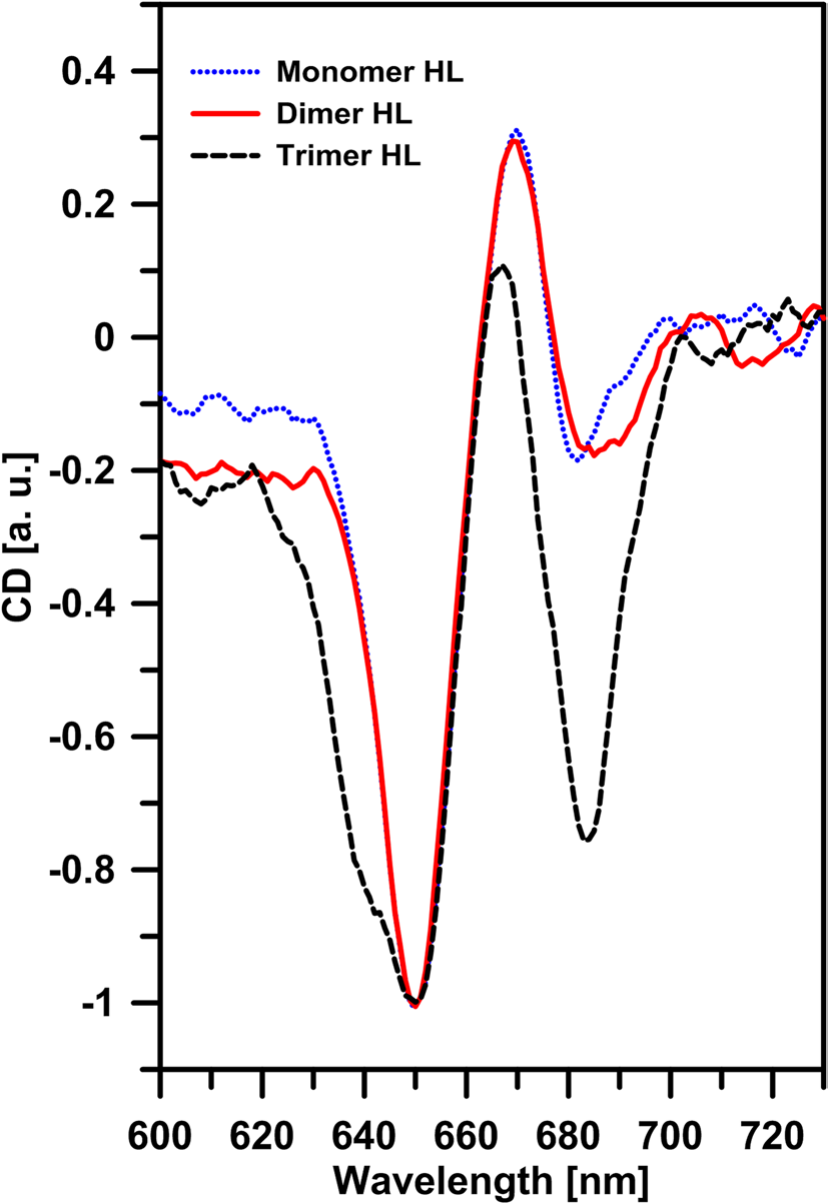
Room temperature CD spectra in the red region of different LHCII forms. The spectra were measured from the LHCII complexes separated by means of the non-denaturing gel electrophoresis. During separation the LHCII trimers and monomers mixture was illuminated with high light intensity (HL, 1200 µmol photons m^−2^ s^−1^). The spectra were registered from the complexes located in polyacrylamide gel. Spectra are normalized in the negative maximum at 650 nm

Having in mind the observed regularity according to which illumination causes the disassembly of supramolecular structures formed with LHCII monomers, one can postulate that dimeric structures formed under higher light conditions are different from those observed at lower light conditions. In order to test such a possibility, we have analyzed Chl *a* fluorescence lifetimes, in situ (in gel, see Fig. 2), in dimeric forms of LHCII, separated at different light intensities. The results of such an analysis are presented in Fig. 4, along with fluorescence lifetimes recorded for LHCII trimers and monomers obtained by electrophoresis. The results show amplitudes of fluorescence lifetime components versus amplitude-weighted average fluorescence lifetimes of high light, low light, and zeaxanthin-induced dimers. In the presented consideration the amplitude-weighted average fluorescence lifetime values were used owing to the fact that biological systems characterized by heterogeneity of the fluorophores and excitation energy transfer should be described by an amplitude-weighted average fluorescence lifetime rather than by an intensity-weighted average fluorescence lifetime (Sillen and Engelborghs 1998). Each fluorescence lifetime value in panel c was registered from an individual LHCII dimeric sample obtained with use of native electrophoretic separation. For comparison, mean values of amplitudes of fluorescence lifetime components in relation to the mean values of amplitude-weighted fluorescence lifetime of LHCII monomer (1.85 ± 0.14 ns) and trimer (2.76 ± 0.06 ns) are indicated (panel a and b). Intensity-weighed fluorescence lifetime of studied LHCII forms (*τ*_in_ = 2.82 ± 0.08 ns for monomers and *τ*_in_ = 3.50 ± 0.08 ns for trimers) are very close to the values which were previously reported for LHCII trimers and monomers located in a detergent environment [polyacrylamide gel strips or detergent solution (Ilioaia et al. 2008; Janik et al. 2015, 2016; Moya et al. 2001; van Oort et al. 2007)]. As can be seen, the amplitude-weighted average fluorescence lifetimes determined for LHCII dimers, are variable in a relatively broad range, in contrast to monomers and trimers. The average fluorescence lifetime values are highest (1–2 ns) in the case of dimeric forms of LHCII which are formed under the presence of exogenous zeaxanthin (Zea) which has been reported to destabilize trimeric structures (Janik et al. 2016). The shorter lifetimes (~1 ns) are observed for the fractions of dimeric LHCII obtained during electrophoretic separations under illumination with low light. Importantly, the lowest (0.6–0.9 ns) average fluorescence lifetimes are observed in the samples illuminated with high light. To determine the contribution of each component in the fluorescence lifetime for each LHCII dimeric form, fluorescence decay kinetics were fitted with three lifetime components: *τ*_1_ = 3.7 ns, *τ*_2_ = 1.8 ns, *τ*_3_ = 0.3 ns. Fluorescence decay kinetics of low light or zeaxanthin-induced dimer forms deconvoluted using the above components had a χ^2^ ~ 1. The high light-induced dimers deconvoluted with the same components had a χ^2^ ~ 1.5. The best fit for high light-induced dimers with χ^2^ ~ 1 was achieved using the following components: *τ*_1_ = 2.5 ns, *τ*_2_ = 1.0 ns, *τ*_3_ = 0.2 ns, as shown in Fig. S5. Figure 4c presents the distribution of the amplitudes of the lifetime components in different dimeric LHCII forms. The amplitude of the shortest lifetime component (0.3 ns) increased from 41 ± 11% in zeaxanthin-induced dimers to 74 ± 3% in high light-induced dimers. Such an observation and shortening of the average fluorescence lifetime of the LHCII sample show that the high light-induced dimer is characterized by spectral forms responsible for excitation quenching. The dependence of LHCII dimer lifetime values on light intensity, compared to monomers and trimers, indicates that monomers in dimeric assemblies are in a different configuration than that known in trimers, additionally characterized by a high rate of excitation quenching. Strong support to such a conclusion comes from the spectroscopic fluorescence emission analysis (see Fig. 5). As can be seen, the dimers formed under elevated light intensities, are characterized by the presence of low energy spectral forms which give rise to a higher fluorescence level in the long-wavelength spectral region (700–760 nm). Moreover, the relatively low in intensity, separate emission band in the region of 650 nm is observed. This emission band, as it was shown earlier (Peterman et al. 1996; Janik et al. 2015), is characteristic of LHCII monomers and is absent in assembled trimeric structures. Fluorescence emission at 650 nm in the LHCII monomers can be assigned to Chl *b*, resulting from the uncoupling of the excitation energy transfer between Chl *b* and Chl *a* located in adjacent monomers, during light-induced LHCII trimers monomerization (Janik et al. 2015). Certainly, one should exclude the possibility that fluorescence emission from Chl *b*, observed in high light-induced dimers, is not a symptom of photodamage caused by strong light intensities applied during electrophoresis. For this purpose, 77 K fluorescence emission spectra of dark-adapted or high light-treated LHCII monomers were measured (Fig. S6). Certainly, it must be noted that precise determination of the exciton pathway is not possible without detailed multi-wavelength fluorescence lifetime analysis (Miloslavina et al. 2008; Kanazawa et al. 2014; Unlu et al. 2014). The emission spectra for the system studied matched that of LHCII monomers. Moreover, no increase in fluorescence emission in the spectral region of 650 nm was observed for LHCII monomers obtained by electrophoretic separation under high light (1843 µmol photons m^−2^ s^−1^) compared to that of monomers electrophoresed in control dark conditions. This may be interpreted as an indication of no damaged antenna pigment–protein complexes with uncoupled Chl *b* to Chl *a* excitation energy transfer under high light illumination. Thus, a different configuration of molecular assembly of dimers formed under high light as compared to the dimers formed under low light can be concluded. Distinctive molecular organization of energy-quenching dimers can be also deduced on the basis of the circular dichroism (CD) spectra analyzed in the Q spectral region (Fig. 6). The CD spectrum of high light-treated LHCII trimers obtained by native electrophoresis in the described experiment is similar to the spectrum of quenched LHCII trimers located in a polyacrylamide gel described by Ilioaia and coworkers (Ilioaia et al. 2008). The CD spectrum of high light-illuminated monomers presented in Fig. 6 is characterized by a lack of most of the negative signal at 680 nm in comparison to non-illuminated monomers (spectrum showed in Fig. S7). Similar change in the CD spectrum of LHCII monomers isolated from samples treated with 1000 µmol photons m^−2^ s^−1^ was previously observed (Garab et al. 2002). In the Q spectral region, excitonic states of Chl *a* and Chl *b* display bands at (−)650, (+)670 and (−)682 nm. As can be seen, the dimeric forms characterized by excitation quenching present distinctive differences at 690 nm as compared to LHCII monomers and trimers treated with high light intensity (Fig. 6).

**Fig. 7:**
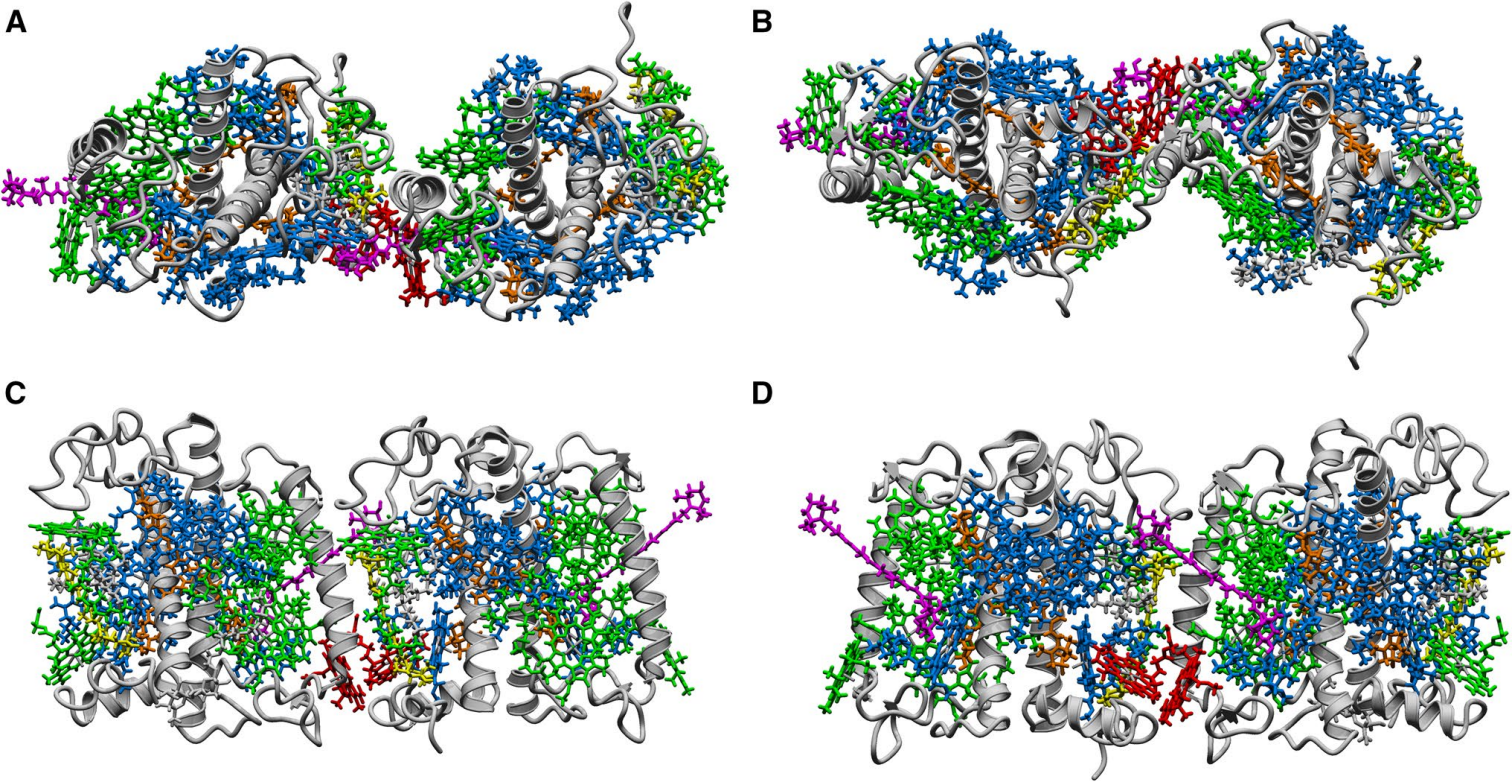
Proposed graphical presentation of high light-induced dimer structure created using YASARA (Krieger et al. 2002). **a** Top view (from the stromal side), **b** bottom view (from the lumenal side), **c, d** side views from the membrane plane perspectives. For clarity of presentation, interacting Chl *a* and *b* are marked *red*. Distance between Mg^2+^ ions in marked chlorophylls is equal to 0.906 nm. The illustration is based on the crystal structure of LHCII from PDB (ID: 2BHW). *blue* Chl *a, green* Chl *b, yellow* violaxanthin, *magenta* neoxanthin, *orange* lutein

It is well known that quenching of energy is attributed to specific pigment arrangement in a quenching structure of LHCII complex in comparison to efficient light-harvesting LHCII forms. The average center-to-center distance between two neighboring chlorophylls for efficient light transfer in LHCII is above 1 nm (Liu et al. 2004). As it was presented earlier, photoprotective energy dissipation is associated with Chl–Chl close contact (<1 nm) and specific mutual chlorophylls ring orientation (Liu et al. 2004; Standfuss et al. 2005; Duffy et al. 2008). Figure 7 presents a proposition for the LHCII dimeric structure, in which two peripheral chlorophyll molecules: Chl 14 and Chl 8 (according to nomenclature in (Standfuss et al. 2005) Chl *b* and Chl *a*, respectively), which are natively separated by a significant distance within a single LHCII monomer, are placed relatively close to each other (less than 1 nm). In our opinion, this newly forming pair of chlorophyll molecules, when the said LHCII dimer is created, may be considered as a possible candidate for energy-quenching trap. Only these two chlorophylls, when two monomers interact at a close distance, may give rise to low energy excitonic or charge transfer bands which can potentially act as energy traps relevant for photoprotection. Certainly, it has to be emphasized that the determination of exact molecular structure of energy-quenching LHCII dimers is only possible on the basis of specially addressed structural biology experiments. Thus, the role of Chl 14 and Chl 8 pair in energy quenching may be only discussed in terms of a research hypothesis.

It has been previously argued that LHCII aggregation is connected with efficient excitation energy quenching what was examined in isolated LHCII samples (Horton et al. 1991; Johnson and Ruban 2009b; Miloslavina et al. 2008) as well as in the thylakoid membrane (Yoshioka-Nishimura and Yamamoto 2014; Belgio et al. 2012; Tian et al. 2015). The quenched state of such aggregates was associated with distinctive rise in a 77 K fluorescence emission band at 700 nm. As can be seen, the fluorescence emission spectrum of the high light-induced dimers is not characterized by an intensive long-wavelength emission band. On the other hand, it should be kept in mind that emission spectra showed in this report were measured from LHCII dimers located in a detergent solution which prevents molecules aggregating. Additionally, it should be taken into consideration that lifetime values of LHCII complexes determined in a detergent solution are longer than that in vivo (Belgio et al. 2012; Tian et al. 2015). Nevertheless, average fluorescence lifetime values of dimers formed under high light treatment are the shortest among the lifetime values of the other LHCII forms studied in a detergent solution (Fig. 4). This shows that the dimers formed de novo out of monomers and characterized by different molecular organization than dimers formed via dissociation of a single monomer from the trimeric structure, can effectively quench excitations, as manifested by a quenching of fluorescence. Having in mind that aggregated LHCII monomers are more efficient excitation quenchers than aggregated trimers (van Oort et al. 2007), LHCII dimers are believed to contribute in effective energy quenching when LHCII antenna switch from light harvesting to energy dissipation activity. The data presented in this report demonstrate that light-induced LHCII monomerization in vivo (Garab et al. 2002; Bielczynski et al. 2016; Janik et al. 2015) may be followed by formation of dimers able to effectively protect themselves via excessive excitation quenching. On the other hand, it cannot be excluded and actually it is highly probable that LHCII dimers could self-associate and form larger supramolecular structures, e.g., lamellar aggregates or macrodomains (Jennings et al. 1991; Barzda et al. 1996), which are capable of even more efficient excitation quenching under severe light stress conditions.

In summary, a light-induced dimeric fraction of LHCII in a quenched state was found. It is known that LHCII complexes under strong light condition may exist in a different quenching forms e.g., monomers and aggregates. Therefore, the appearance of the LHCII dimers after high light-treated whole spinach leaves or LHCII in a detergent suspension suggests that dimers could be considered as partially responsible for dissipation of excess excitation energy in the thylakoid membrane under strong light conditions. However, it should be taken into consideration that the photoprotective mechanisms in vivo are more complex in their nature. Thus, most likely, LHCII dimerization could act in parallel and in aid with multiple other energy dissipation processes protecting the photosynthetic apparatus of plants against light-induced damage.

## Electronic supplementary material

Below is the link to the electronic supplementary material.

Supplementary material 1 (DOCX 1312 KB)
