# Supplementary material for: Light-induced formation of dimeric LHCII
Source: Photosynth Res. 2017 Apr 19;132(3):265–76. doi: 10.1007/s11120-017-0387-6 (PMC5443882; doi:10.1007/s11120-017-0387-6)
Supplement: Supplementary file 1 — Supplementary material 1 (DOCX 1312 KB) [file 11120_2017_387_MOESM1_ESM.docx]

**Supplemental Materials**

**Light-induced formation of dimeric LHCII**

Ewa Janik^a,b,*^, Joanna Bednarska^a,c^, Karol Sowinski^a,d^, Rafal Luchowski^a^, Monika Zubik^a,e^ Wojciech Grudzinski^a^ and Wieslaw I. Gruszecki^a,*^

1. Department of Biophysics, Institute of Physics, Maria Curie-Sklodowska University, Pl. Marii Curie-Sklodowskiej 1, 20-031 Lublin, Poland
2. Department of Cell Biology, Institute of Biology and Biochemistry, Maria Curie-Sklodowska University, ul. Akademicka 19, 20-033 Lublin, Poland
3. Department of Medicine, Imperial College London, Du Cane Road, London W12 0NN, United Kingdom
4. Present address: Chair and Department of Synthesis and Chemical Technology of Pharmaceutical Substances Faculty of Pharmacy, Medical University, Chodzki 4a, 20-093 Lublin, Poland
5. Department of Metrology and Modelling of Agrophysical Processes, Institute of Agrophysics of Polish Academy of Sciences, Doswiadczalna 4, 20-290 Lublin, Poland

**
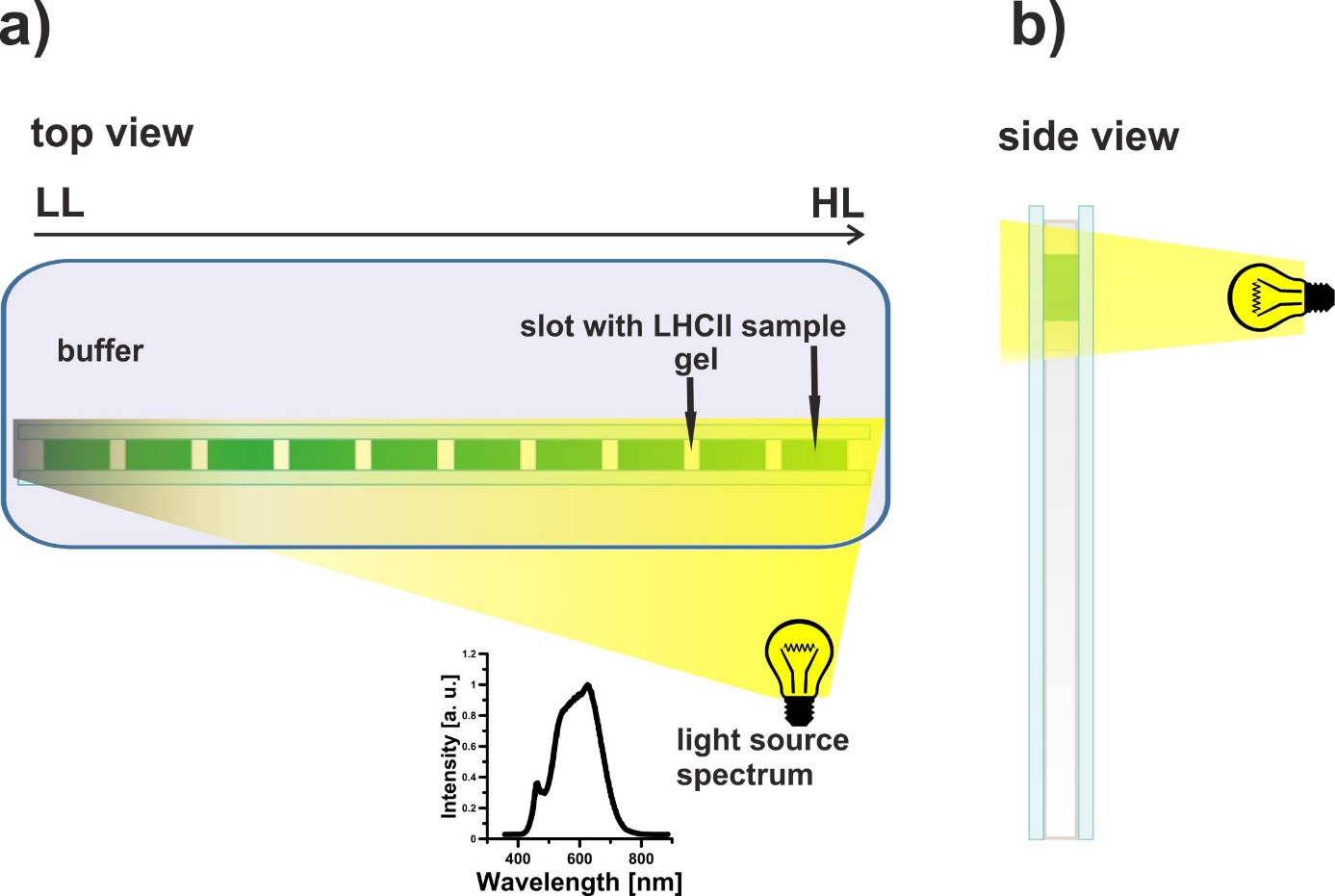
**

**Fig. S1.** Scheme depicting the method of illumination of pigment-protein complex samples during the electrophoretic separation. In the figure, top view (a) and side view (b) of graphical presentation of an electrophoretic set is displayed. Each slot containing the sample was illuminated with different light intensity (from ~10 to ~1200 µmol photons m^-2^s^-1^). It was achieved by gel illumination in an intensity gradient of a LED light source. Light intensity in each slot was precisely measured using a photometer, directly before each experiment.

**
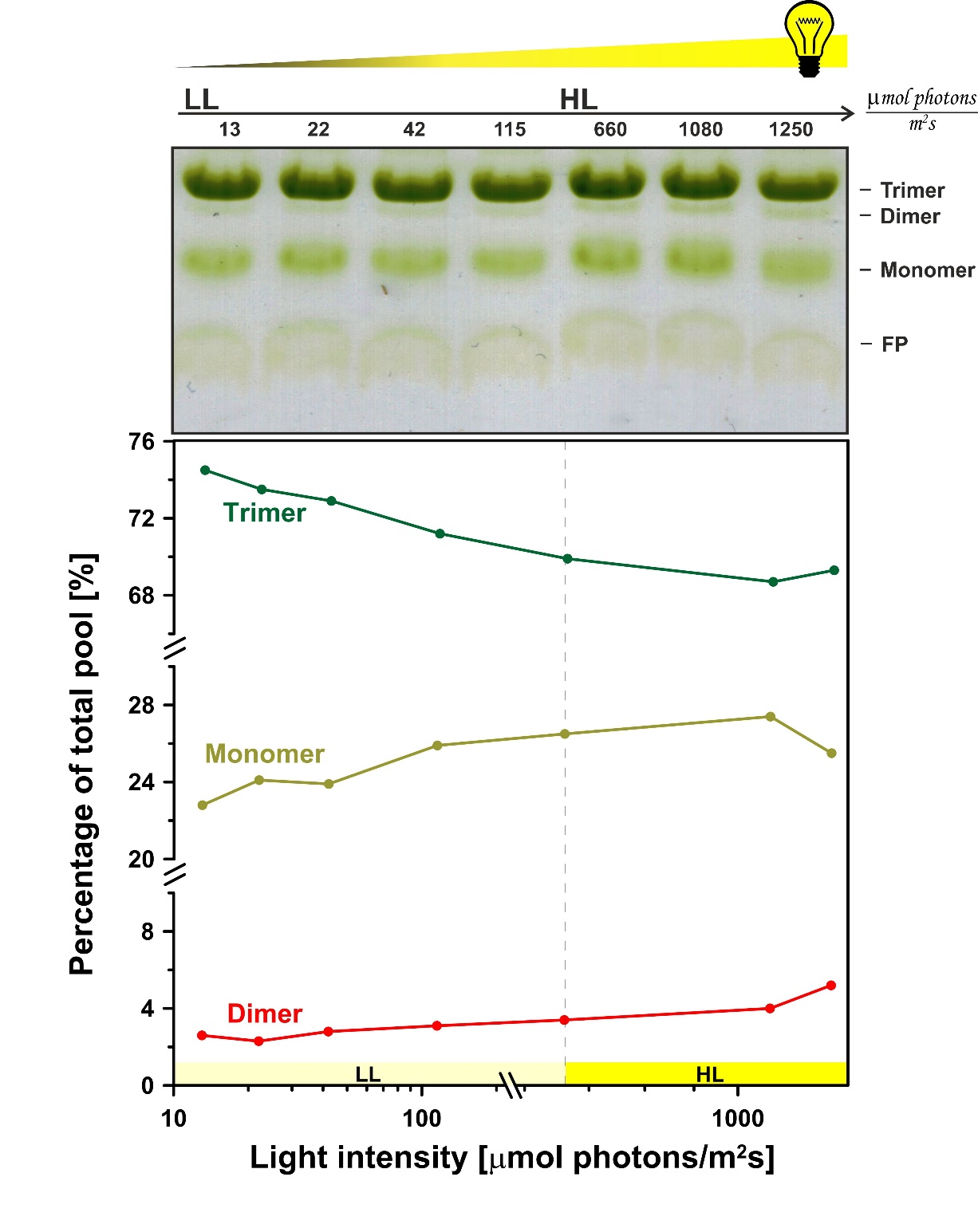
**

**Fig. S2.** Electrophoretic analysis of LHCII oligomeric forms in LHCII samples illuminated with different light intensities. LHCII was suspended in 0.1 % DM. Electrophoresis was conducted under constant illumination starting from the sample loading (as described in the main part of the paper). Each slot was illuminated with different light intensity (from 13 µmol photons m^-2^s^-1^ to 1250 µmol photons m^-2^s^-1^). It was achieved by gel illumination in an intensity gradient of a LED light source (as it is schematically presented in the Figure S1). Light intensity in each slot was precisely measured using a photometer before each experiment. Upper panel shows exemplary results of the electrophoretic gel analyses with band assignment (FP, free pigment fraction). Lower panel presents the quantity analysis of LHCII trimers, dimers and monomers in the samples. Relative intensities of the bands corresponding to different LHCII forms were quantified by scans analyzed with ImageJ2x software.


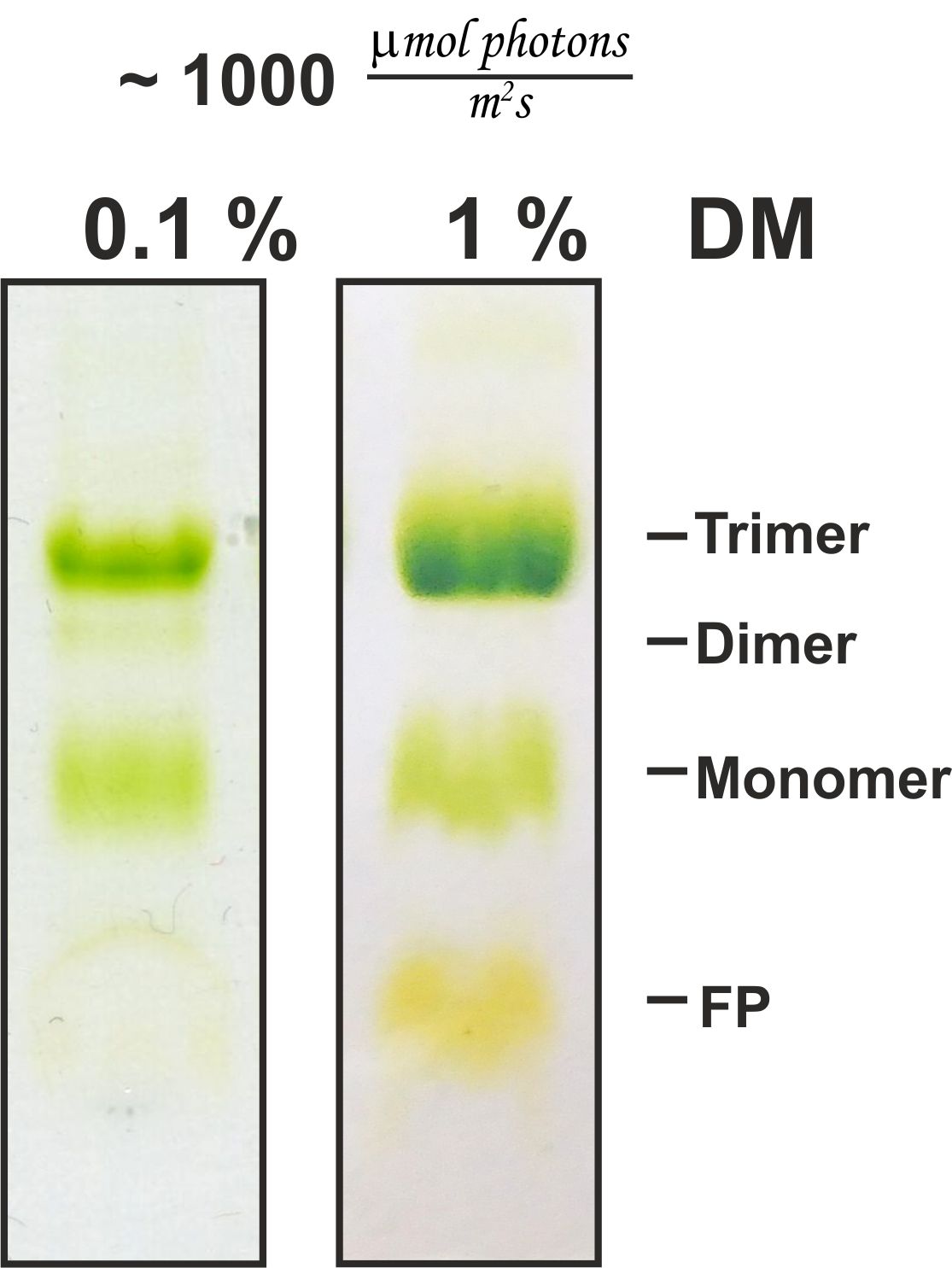


**Fig. S3.** LHCII forms in different detergent concentrations. Electrophoretic analysis of LHCII oligomeric forms in LHCII samples illuminated with high light intensity (~ 1000 µmol photons m^-2^s^-1^). LHCII was suspended in 0.1 % or 1 % DM. Electrophoresis was conducted under constant illumination starting from the sample loading (as described in the main part of the paper). It was achieved by gel illumination in an intensity gradient of a LED light source (as it is schematically presented in the Figure S1). Light intensity in each slot was precisely measured using a photometer before each experiment.

**
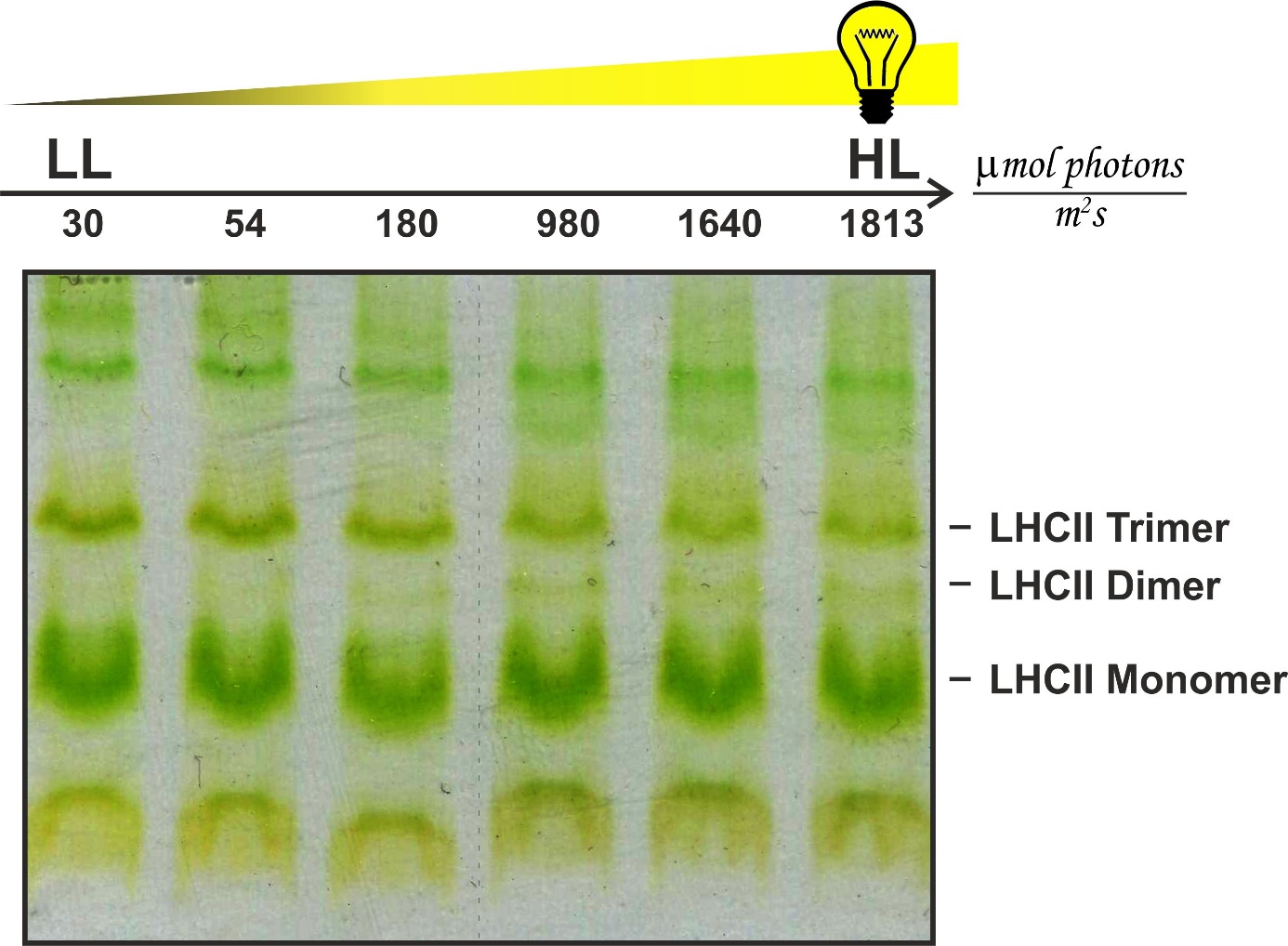
**

**Fig. S4**. Electrophoretic analysis of pigment-protein complexes in the thylakoid sample illuminated with different light intensities. Thylakoids were isolated according to (Garstka et al. 2005) and next suspended in 0.1 % DM. Electrophoresis was conducted under constant illumination starting from the sample loading (as described in the main part of the paper). Each slot was illuminated with different light intensity (from 30 µmol photons m^-2^s^-1^ to 1813 µmol photons m^-2^s^-1^). It was achieved by gel illumination in an intensity gradient of a LED light source (as it is schematically presented in the Figure S1.). Light intensity in each slot was precisely measured using a photometer before each experiment.


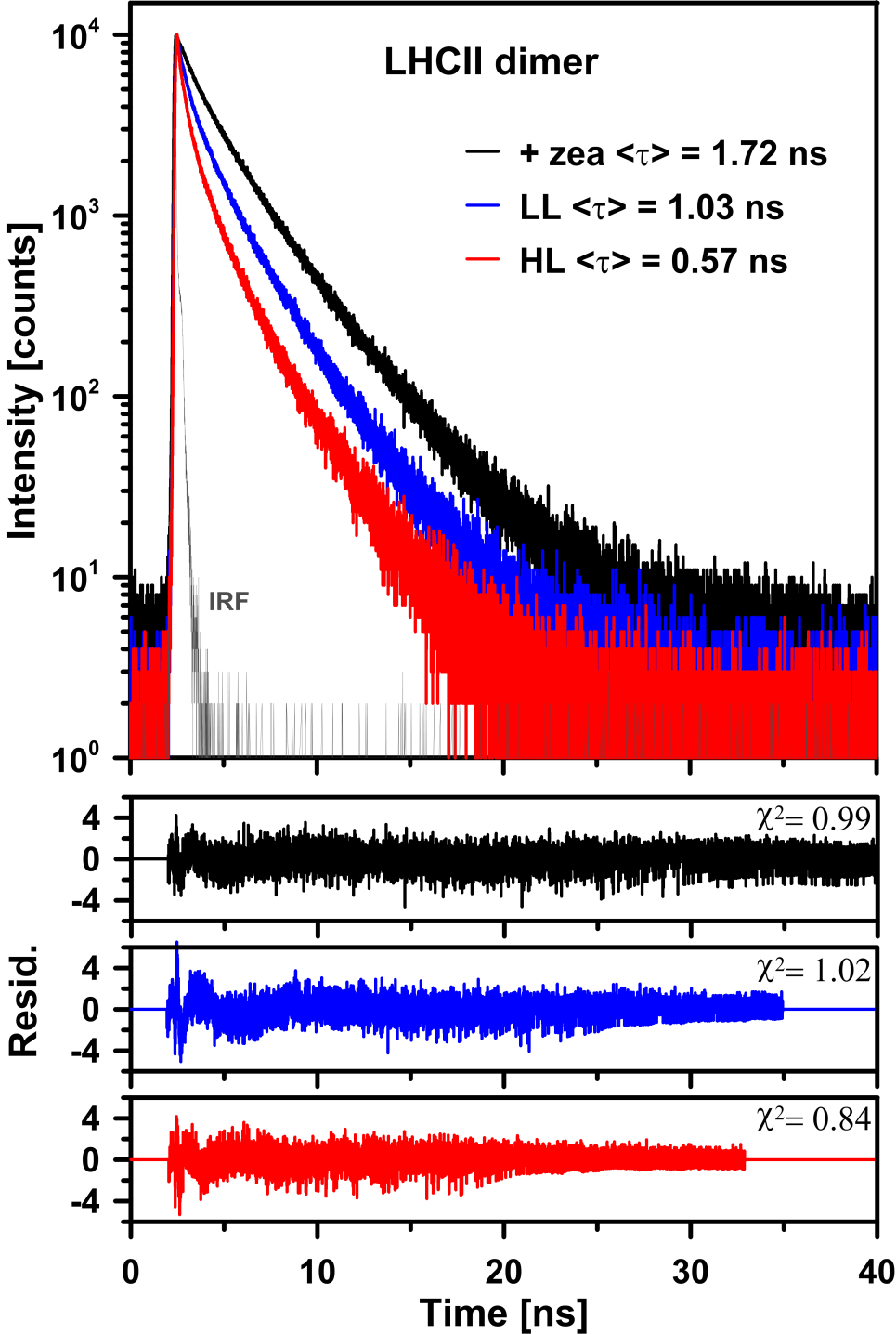


**Fig. S5.** Fluorescence decay traces of Chl *a* emission in dimeric LHCII forms. Fluorescence decay curves were recorded directly after electrophoresis from LHCII complexes located in polyacrylamide gel (as described in the main part of the paper). Excitation and detection were set at 470 nm and 680 nm, respectively. Fluorescence intensity decays were analyzed by reconvolution with the instrument response function and analyzed as a sum of exponential terms with FluoFit Pro v 4.5.3.0 (PicoQuant, Germany). The quality of the fit was judged by the χ^2^ value. The fluorescence decay kinetics were fitted with three components characterized by the following lifetimes: τ_1_= 3.7 ns, τ_2_= 1.8 ns and τ_3_= 0.3 ns in the case of the LHCII dimers induced by exogenous zeaxanthin or low light intensity (16 µmol photons m^-2^s^-1^), and τ_1_ = 2.5 ns, τ_2_ = 1.0 ns and τ_3_ = 0.2 ns in the case of the LHCII dimers induced by high light intensity (1240 µmol photons m^-2^s^-1^). The procedure of preparation of the zeaxanthin or light-induced LHCII dimers is described in the main part of the publication. Typical fluorescence lifetime decays, amplitude-weighted average lifetime <τ> values, χ^2^ values and residuals of the curve fit are displayed in the figure.


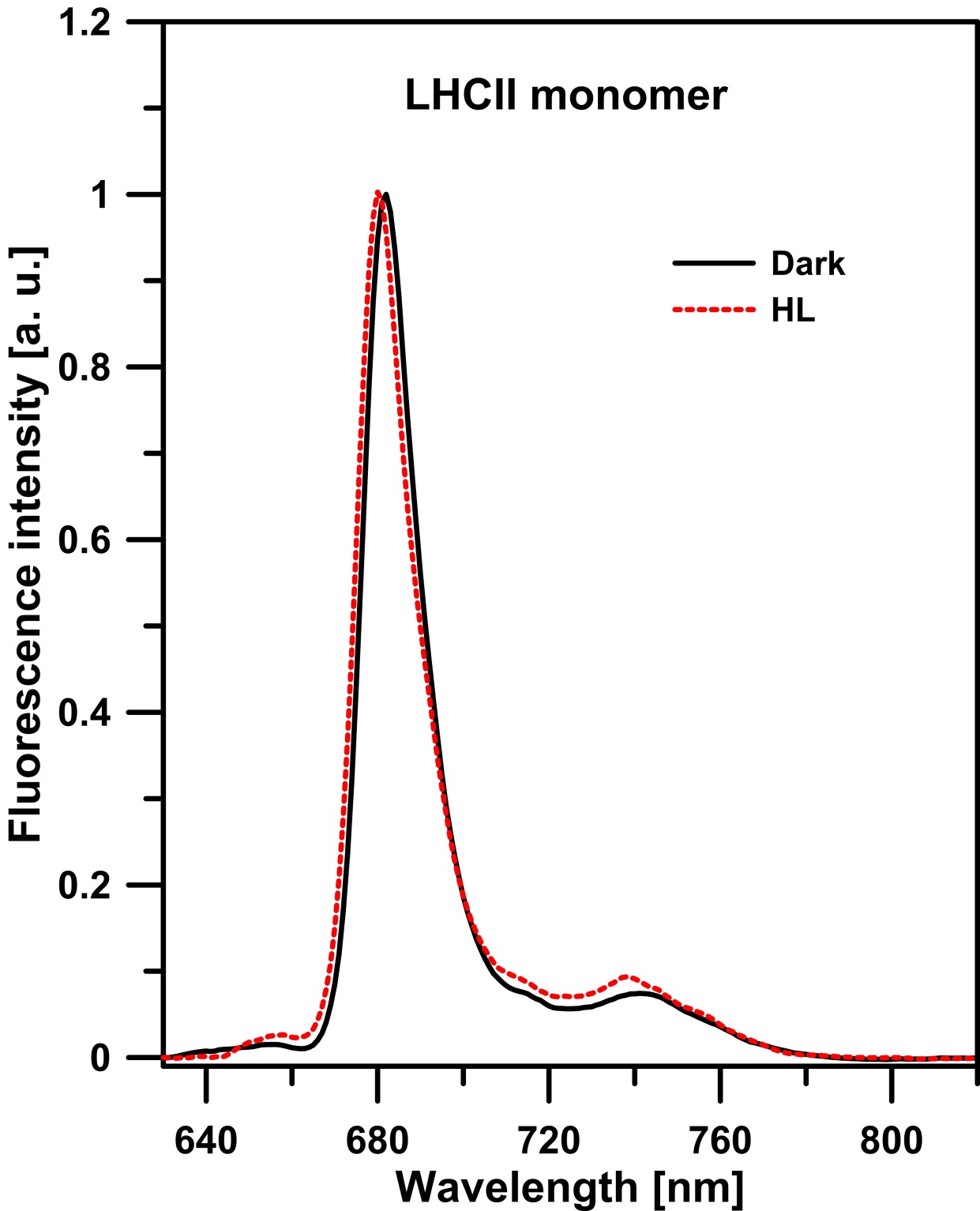


**Fig. S6.** 77 K Chl *a* fluorescence emission spectra of the LHCII monomers. Monomers were obtained by electrophoretic separation of the LHCII complexes isolated from the dark-adapted spinach leaves and next solubilized in 0.1% DM solution. Electrophoresis was done in the dark (dark, black solid line) or under the electrophoretic gel illumination with light intensity of 1200 µmol photons m^-2^s^-1^ (HL, red dashed line) during the separation (details described in the main part of the publication). The spectra were normalized in the maximum. Spectra were measured from the LHCII complexes located in polyacrylamide gel.


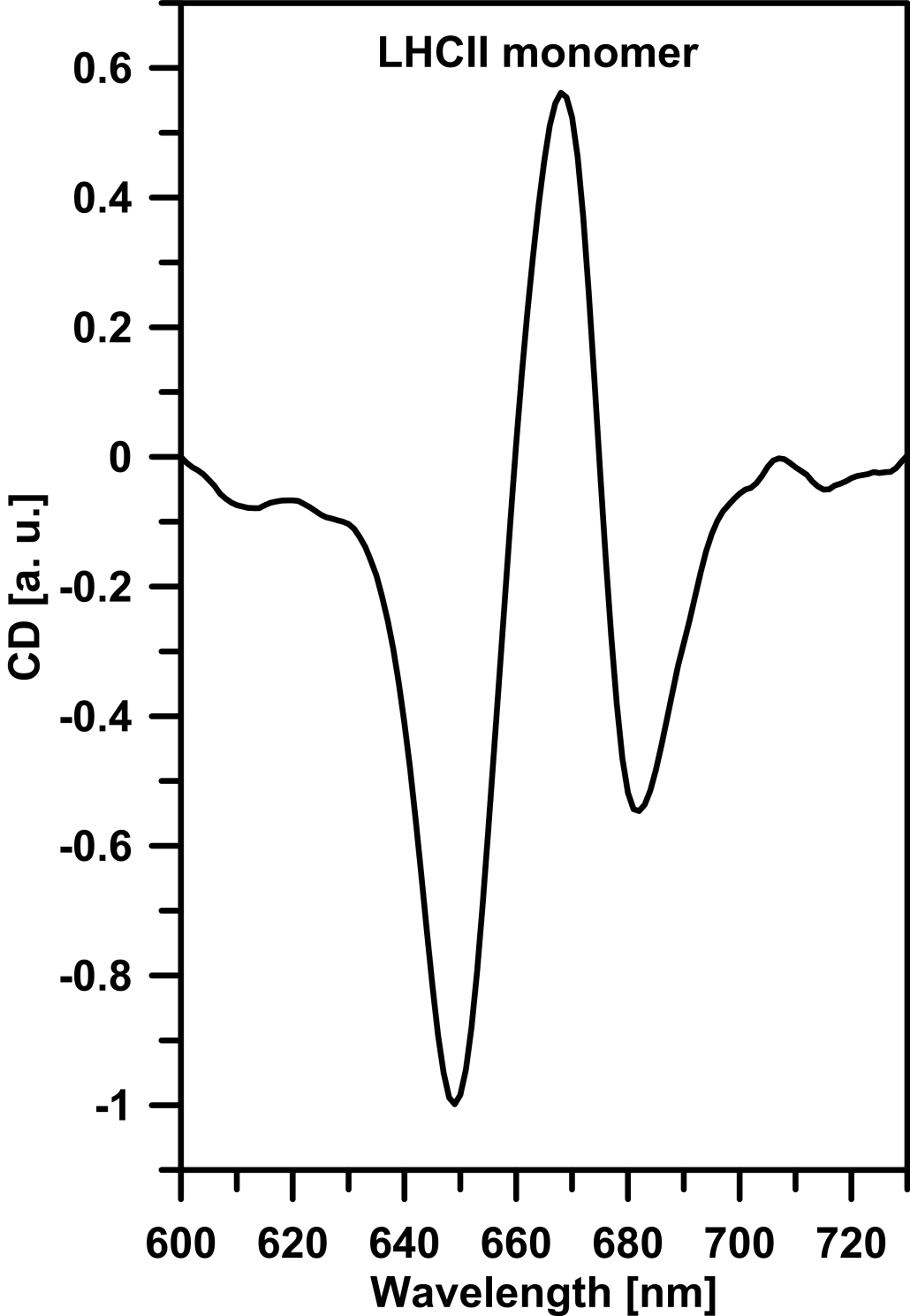


**Fig. S7.** Room temperature CD spectrum in the red region of LHCII monomers. The spectrum was measured from the LHCII complexes separated by means of the non-denaturing gel electrophoresis and located in polyacrylamide gel. Electrophoresis was run in the dark. Spectrum is normalized in the negative maximum at 650 nm. The methods of the CD spectrum measurement is described in the main part of the paper.

**Garstka M, Drozak A, Rosiak M, Venema JH, Kierdaszuk B, Simeonova E, van Hasselt PR, Dobrucki J, Mostowska A (2005) Light-dependent reversal of dark-chilling induced changes in chloroplast structure and arrangement of chlorophyll-protein complexes in bean thylakoid membranes. Biochim Biophys Acta 1710 (1):13-23. doi:S0005-2728(05)00193-3 [pii]10.1016/j.bbabio.2005.08.006**
